# Cloud-controlled microscopy enables remote project-based biology education in underserved Latinx communities

**DOI:** 10.1016/j.heliyon.2022.e11596

**Published:** 2022-11-15

**Authors:** Pierre V. Baudin, Raina E. Sacksteder, Atesh K. Worthington, Kateryna Voitiuk, Victoria T. Ly, Ryan N. Hoffman, Matthew A.T. Elliott, David F. Parks, Rebecca Ward, Sebastian Torres-Montoya, Finn Amend, Natalia Montellano Duran, Paola A. Vargas, Guadalupe Martinez, Sandra M. Ramirez, Lucia Elena Alvarado-Arnez, Drew Ehrlich, Yohei M. Rosen, Arnar Breevoort, Tallulah Schouten, Sri Kurniawan, David Haussler, Mircea Teodorescu, Mohammed A. Mostajo-Radji

**Affiliations:** a Department of Electrical and Computer Engineering, University of California Santa Cruz, Santa Cruz, CA, 95064, USA; b Genomics Institute, University of California Santa Cruz, Santa Cruz, CA, 95060, USA; c Live Cell Biotechnology Discovery Lab, University of California Santa Cruz, Santa Cruz, CA, 95060, USA; d Department of Microbiology and Environmental Toxicology, University of California Santa Cruz, Santa Cruz, CA, 95064, USA; e Department of Molecular, Cellular and Developmental Biology, University of California Santa Cruz, Santa Cruz, CA, 95064, USA; f Institute for the Biology of Stem Cells, University of California Santa Cruz, Santa Cruz, CA, 95064, USA; g Department of Biomolecular Engineering, University of California Santa Cruz, Santa Cruz, CA, 95064, USA; h Alisal High School, Salinas, CA, 93905, USA; i Biotechnology, Universidad Catolica Boliviana San Pablo, Santa Cruz de la Sierra, Bolivia; j National Research Coordination, Franz Tamayo University, La Paz, Bolivia; k Department of Computational Media, University of California Santa Cruz, Santa Cruz, CA, 95064, USA; l Department of Neurology, University of California San Francisco, San Francisco, CA, 94158, USA; m Howard Hughes Medical Institute, University of California Santa Cruz, Santa Cruz, CA, 95064, USA

**Keywords:** STEM education, Cloud laboratories, Remote education, Biology education, Latinx, Hispanics, Undeserved communities

## Abstract

Project-based learning (PBL) has long been recognized as an effective way to teach complex biology concepts. However, not all institutions have the resources to facilitate effective project-based coursework for students. We have developed a framework for facilitating PBL using remote-controlled internet-connected microscopes. Through this approach, one lab facility can host an experiment for many students around the world simultaneously. Experiments on this platform can be run on long timescales and with materials that are typically unavailable to high school classrooms. This allows students to perform novel research projects rather than just repeating standard classroom experiments. To investigate the impact of this program, we designed and ran six user studies with students worldwide. All experiments were hosted in Santa Cruz and San Francisco, California, with observations and decisions made remotely by the students using their personal computers and cellphones. In surveys gathered after the experiments, students reported increased excitement for science and a greater desire to pursue a career in STEM. This framework represents a novel, scalable, and effective PBL approach that has the potential to democratize biology and STEM education around the world.

## 1 Introduction

Given the rapid rise in demand for workers in science, technology, engineering, and mathematics (STEM), there is a growing need for high-quality STEM education. However, access to STEM education remains highly unequal. In the United States, Latinx people are the fastest growing demographic, currently encompassing over 19% of the country's population while receiving less than 12% of degrees awarded in STEM fields [1], [2].

Access to quality education has further deteriorated during the COVID-19 pandemic [3]. The migration from in-person classes to remote video conferencing systems like Zoom has presented many challenges for students and educators [4]. The negative impacts of this migration have disproportionately affected students from traditionally underrepresented groups [5], [6]. This unequal learning loss, particularly in STEM disciplines, has not only widened the gap between students but has jeopardized global initiatives such as Education 2030 and the United Nations Sustainable Development Goals [7], [8]. New approaches for high-quality remote STEM education hold the potential to reverse this trend and further spread access to educational resources worldwide.

Project-based learning (PBL) is an effective approach for teaching complex STEM concepts [9], [10], particularly for students from backgrounds typically underrepresented in STEM professions [1], [11], [12], [13]. In PBL, students have the chance to learn in a hands-on matter, investigating deeper questions and discovering truth for themselves [1], [13], [14]. Despite the benefits of PBL, three significant barriers stand in the way of its broad implementation into STEM laboratory courses: 1) high local infrastructural cost required to perform complex PBL projects, 2) limited teacher training, and 3) potential exposure to hazardous materials [1], [15]. The additional challenges of remote learning further compound these barriers. The lack of an effective replacement for in-person lab activities has caused sub-par learning experiences for students. Finding a way to provide students with the experience of practical project-based lab work is essential if we expect remote classes to work as well as in-person ones.

Several systems have been proposed to address this issue by facilitating at-home PBL. Different initiatives and companies have created do-it-yourself (DIY) kits that students can use to perform simple experiments [16], [17], [18], [19]. While intriguing, these kits require the acquisition and shipping of individual kits to each student, group of students, or school [20], [21] and are therefore not scalable, nor can they easily reach isolated communities. Another approach involves creating experiment protocols involving common household items [22], [23]. This approach is limited in terms of what experiments can be run and assumes access to a common set of objects. And then there is the simulation approach, wherein students interact with a video game that attempts to replicate the results of a real experiment [24], [25]. While clever, these solutions only enable students to explore a few canned options with a predetermined “right answer,” denying them the true experience of scientific experimentation and discovery.

It has also been shown that classroom examples that factor in local issues lead to better student engagement and learning outcomes, particularly for students from underrepresented backgrounds [1], [26], [27], [28]. This is hard for simulations and “kitchen chemistry” kits to do. Therefore, an approach that is scalable at a low cost, adaptable to local contexts, and easily accessible to students has the potential to revolutionize STEM education by democratizing PBL implementation in classrooms globally.

The Internet of Things (IoT) has transformed many fields of society and research, including agriculture [29], healthcare [30] and wildlife conservation [31]. Yet, the adoption of IoT in the classroom has been minimal [32]. When applied to the remote operation of lab equipment, IoT can be used to facilitate educational lab experiences. Early work using internet-connected single camera microscopes used photophobic organism samples and enabled remote control of light stimulation to create interactive experiments [33], [34]. The activities enabled by these devices are short and generally designed to fit in the time frame of a single class session. Programs like this provide a proof of principle for the usability of IoT technologies in education. However, these technologies are not designed to be adaptable for context-informed PBL and running experiments requiring multiple conditions over longer multi-day periods. One recent study used an IoT system in a longer-term experiment. Students remotely monitored soil moisture while evaluating the effects of ground cover on plant seedlings [35]. However, this project was not fully remote and required students to make measurements in person at the site of the experiment, and must therefore be confined to a defined geographical location.

When considering the technologies needed to set up a fully remote, longer-term experiment, remote microscopy is alluring in its simplicity. Simply point a camera at a phenomenon, and people can make observations from anywhere. Existing microscope systems can be modified with widely available internet-connected cameras, or purpose-built connected imaging systems can be developed. Unlike systems that involve direct physical interaction with samples, remote imaging systems are non-invasive and capable of being set up at a low cost. Imaging systems capable of performing near real-time simultaneous longitudinal tracking of cells, 3D cultures, and small organisms have started to emerge in research settings [36], [37], [38]. These systems can be used to perform experiments with a diversity of models and conditions. The rapid cloud-based transfer and storage of data and their relatively simple interfaces make them excellent tools for science [37], [38]. Given the versatility of these devices, applying them to remote education represents a cost-effective and scalable approach to performing PBL.

Here we describe the implementation of these technologies in the biology classrooms of Latinx communities in the United States and Latin America using examples relevant to their local surroundings. We demonstrate the capability of this framework to perform a single shared experiment for several groups around the world simultaneously. Furthermore, we provide a framework for interested parties to remotely perform their context-informed PBL-based courses.

To evaluate this program's utility, scalability, and impact, we ran several iterations of the PBL-based remote class with different user groups. We conducted user surveys with two of the participant groups: high school students in California's Central Valley and college students in Bolivia. We compared the user experiences of both groups and contrast them with previous work performing in-person PBL with similar cohorts [1]. We show that IoT-enabled remote PBL is an effective and scalable approach for serving underrepresented students in STEM and provides a novel platform for comparative experimental STEM education studies.

## 2 Methods

### 2.1 Imaging system selection

Any IoT-based imaging for remote experimental education needs to allow the capture of longitudinal data from the sample on an appropriate timescale. The system should make this data readily available for the students and other users, ideally allowing them to access it from their own devices. Finally, the data should be easily interpretable and permissible for analysis. In the six studies we ran (further described in section 3), we used a system called the “Picroscope” in all of them an additional system called the “Streamscope” in two of them.

**Figure 1:**
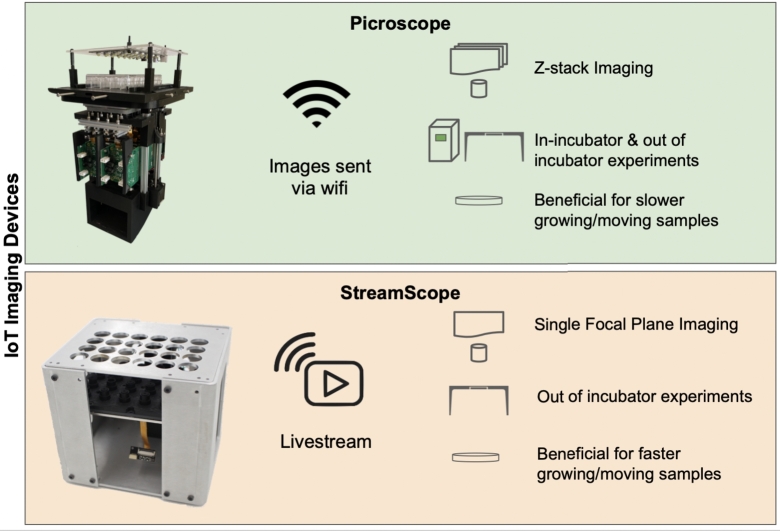
**Comparison of remote imaging devices** 2 different remote imaging platforms were used, the Picroscope collects z-stack image data inside an incubator while the Streamscope livestreams a view of a sample on a lab bench.

The “Picroscope” is a low-cost open-source IoT-enabled remote microscopy device built primarily using off-the-shelf components and 3D printed pieces [36], [37] (Fig. 1). The Picroscope captures 3D z-stack data on long timescales and makes the image datasets available to students on an image viewer website (described in Supplemental Video 1). It is designed to image cells, tissues, and organisms growing in the wells of a standard 24 well plate. Picroscopes can also be deployed in a standard temperature and humidity-controlled CO_2_ incubator, allowing the use of biological cultures and samples that require that a controlled environment to thrive. The “Streamscope” is another maker microscope that live-streams video from the wells simultaneously (Fig. 1). Students view the live stream through YouTube. The 3D z-stack image data from a Picroscope is well-suited to samples that are adhered to a well plate where growth is effectively visualized on the scale of hours and days. For faster moving samples like zebrafish or other small animals, the z-stack functionality is less useful, and users benefit more from viewing a video stream. When designing a PBL experiment, It is important to select the right system for the available samples or to select appropriate samples for the availale imaging system.

The Picroscope contains 24 cameras imaging in parallel, allowing 24 students to each capture image data from their own well or all students to view all 24 wells. The wells are formatted into a standard 24-well cell culture plate. Images are uploaded to the Internet, where they can be viewed through a public website. Uploads occur immediately after a timestep is captured, giving end users a near real-time experience looking through a virtual microscope. These image datasets are also stored long-term, allowing for experiments to be replayed or loaded into data analysis pipelines. Picroscope experiments can be run inside a standard CO_2_ and humidity-controlled incubator or outside, giving experiment designers flexibility with the samples they choose.

### 2.2 Platform data analysis pipeline

Since high school and early college-level biology classes don't typically have a programming requirement, it is important to provide user-friendly tools for analysis. A balance should be struck between introducing students to working with code and not requiring them to write much code themselves.

In the programs we ran, students completed a learning module on an online Jupyter notebook server [39] where they learned the basic fundamentals of manipulating experimental data in Python. The module provides them with a coding environment, example code, and online video tutorials that they can follow. A facilitator lectures the students and guides them through completing the learning module. At the end of the experiment, students are guided through the analysis of the data they have collected throughout the experiment and then give a presentation on their findings. Microscopy images recorded from the experiment are analyzed on the notebook server, where a Python GUI application for annotating images was created so that students can highlight findings that they consider significant throughout the experiment. For example, students can classify cancer cells and annotate specific phenotypes they believe are changed by the application of drugs.

### 2.3 User studies

Student users were selected through partnerships with different organizations. Students in California were 4th year high school students at Alisal High School, located in Salinas, California. All students were part of an Advanced Placement (AP) Biology course.

Students in Bolivia came from two different universities:

1) First-year students in the Biotechnology degree at the Universidad Catolica Boliviana San Pablo in Santa Cruz de la Sierra, Bolivia performed the experiments in neuroblastoma cell lines.

2) Second-year Biochemistry students at Franz Tamayo University performed the experiments using chlorine dioxide. Franz Tamayo University has 4 campuses in Bolivia: Santa Cruz de la Sierra, La Paz, El Alto and Cochabamba. The students were from all campuses.

The multinational study involving Bolivia, Mexico, Peru, Brazil and Spain consisted of students undertaking a two weekend-long course through the nonprofit organization Science Clubs International.

### 2.4 Remote teaching

Before each course started, we met with the lead instructor and designed the experiments that were relevant to the course. When applicable, we retained the core curricula from the national standards. All supplemental teaching by UCSC researchers was facilitated using Zoom. At Alisal High School, classes took place once a week. For the university students, the classes were taught over a three-week period. The Science Clubs International courses were taught in two sessions, one week apart from each other. Instructors were graduate students at the University of California, Santa Cruz. The courses in Bolivia were taught in Spanish, while the courses in the United States and through Science Clubs International were taught in English.

### 2.5 User demographics

In accordance to local regulations, and in order to maintain students' anonymity, we refrained from obtaining individual identifiable information. All Alisal High School students were aged 17-18, were born in the United States, and self identified as Latinx. All students at the Catholic University of Bolivia self identified as Latinx and were 17-19 years of age. The cohorts were all mixed gender without any overwhelming majority.

### 2.6 Surveys and subject tests

Surveys were conducted using Google Forms; all questions are listed in Supplemental Table 1.

Students at the Catholic University of Bolivia were tested in person using a subject test designed by the course lead instructor. The translation of the test can be accessed in Supplemental Note 6.

### 2.7 Statistical analyses

We confirmed that the data was not normally distributed using the Kolmogorov Smirnov test. Therefore, statistical comparisons between groups of students were conducted using either the Mann-Whitney test (survey question comparisons), or the Wilcoxon Signed Rank test (test score comparisons), which do not assume normal distributions. All significance tests were two-tailed. Significance was established as follows: ⁎=p<0.05; ⁎⁎=p<0.01; ⁎⁎⁎=p<0.001; ⁎⁎⁎⁎=p<0.0001.

### 2.8 Culture of neuroblastoma cells

Mouse neuroblastoma cells (ATCC # CCL-131) were cultured in standard tissue culture incubators at 37 °C in Dulbecco's Modified Eagle's Medium - high glucose (Millipore Sigma # D6429) supplemented with 10% Fetal Bovine Serum (Thermo Fisher Scientific # 26140079).

Drugs used were Retinoic Acid (10 μM, Millipore Sigma # R2625), Neurodazine (2 μM, Millipore Sigma # N6664), Primocin (2 μl/ml, Invivogen # Ant-pm-05).

### 2.9 Zebrafish experiments

Fertilized *Danio rerio* eggs were purchased from Carolina Biological Supply Company (Catalog # 155591) and maintained in media containing 15 mM sodium chloride (Millipore Sigma # S9888), 0.5 mm potassium chloride (Millipore Sigma # P3911), 1 mM calcium chloride dihydrate (Millipore Sigma # 223506), 1 mM magnesium sulfate heptahydrate (Millipore Sigma # 1058822500), 150 μM potassium phosphate monobasic (Millipore Sigma # P5655), 50 μM sodium phosphate dibasic heptahydrate (Millipore Sigma #S9390), 0.7 mM sodium bicarbonate (Millipore Sigma # S5761), and 0.1% methylene blue (Millipore Sigma #M9140).

The following drugs were used in the different user studies: caffeine (10 *μ*M, Millipore Sigma # C0750), ammonium nitrate (10 mg/L and 100 mg/L, Millipore Sigma # 221244), (-)-Nicotine (1%, Millipore Sigma # N3876), and chlorine dioxide (0.1%, 0.5% and 1%, Novatech # R-8039A). Gold and graphene nanoparticles were synthesized in-house and used at concentrations ranging from 10 to 100 nM.

### 2.10 Research ethics

The work described here was approved by the ethical committees of the institutions involved. The Catholic University of Bolivia ethics committee approved the work performed at that institution. In accordance with Bolivian regulations, the Franz Tamayo University research oversight committee approved the work at all 4 campuses of this university. All college students were adults and were given informed consent. The Salinas Union High School District approved the work in Alisal High School. All high school students were given informed consent as part of their registration into the AP Biology course, which was cosigned by their parents.

## 3 Results

### 3.1 A curriculum roadmap for remote PBL

**Figure 2:**
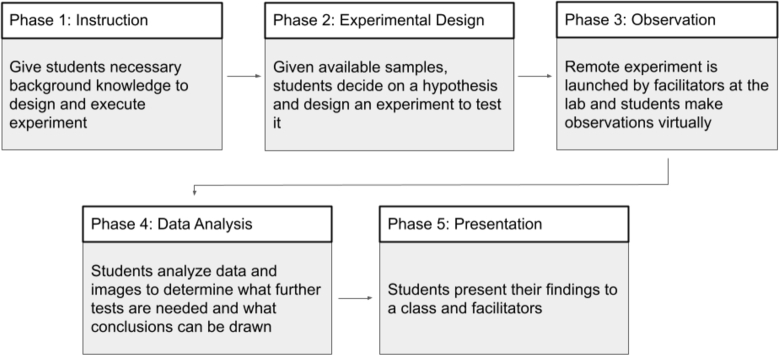
**Program Roadmap:** We developed a program structure with five phases for remote project based supplemental learning.

We defined a roadmap for designing a curriculum program using a remote microscopy system to facilitate project-based experimental biology. The roadmap is aimed at research groups interested in using their lab to facilitate a remote PBL program. The program is designed as a supplement to existing biology courses, and facilitators would be researchers from the lab that wish to participate in educational collaboration with a teacher's class. In the weeks leading up to the remote experiment, the facilitators teach supplemental lessons on various scientific topics. The lessons build on the student's current knowledge and prepare them to design their biological experiment. After the experiment, students analyze their findings and present a conclusion to the class. A broad outline of this framework is shown in Fig. 2.

#### 3.1.1 Phase 1: instruction modules

The first phase of this remote experimental biology course is to provide students with the instruction to support them through designing their experiments, collecting and analyzing data, and coming to scientifically supported conclusions. This is done through a series of lectures and activities in the weeks leading up to the experiment.

Developing a lesson involves first identifying the desired learning outcomes. Desired learning outcomes describe what students should know or be able to do at the end of a lesson. It is useful to clearly articulate the learning outcomes to help inform the students of what to expect from the lesson and gauge their learning. Additionally, they guide the instructor in lesson design. Resources on how to design learning outcomes can be found at [40], [41].

Rather than replicating the content the students are already learning in class, facilitators should aim to show students relevant topics from the perspective of a researcher, with a focus on how to approach a subject from an experimental design standpoint. Complete lesson outlines, learning outcomes, and some examples of activity materials for each lesson can be found in Table 2 and Supplemental Notes 1-5.

#### 3.1.2 Phase 2: experiment design

After facilitators have run lessons presenting the necessary background knowledge, it is time to design the experiment. A good experiment for our platform is one where the sample requires no maintenance after setup, and the observations of interest are all visual. Facilitators introduce the available biological samples and then guide students through defining a testable question and designing an experiment to address that question. The students will then create a proposal outlining their question, experimental design, and hypothesis to be tested. An example activity to help students propose their hypotheses and design their experiment can be found in Supplemental Note 3. In the example proposal, students are given the experimental question “how do certain drugs affect cancer cell growth?”. They then define a hypothesis for their specific drug, determine the dependent variables, independent variables, and controls, and plan out the materials and methods needed to conduct the experiment. With this proposal, the project facilitators will prepare the appropriate samples and set up the remote imaging device. After the setup, the experiment is launched.

#### 3.1.3 Phase 3: observation

Next, students make observations as the experiment runs in real-time. The most substantial hurdle to conducting a successful remote biology course is providing students with the experience of real-time observations of an experiment that does not have a predetermined outcome. This is the essence of doing science, so this issue must be faced. Once the experiment is launched, the device is set up in such a way as to allow students to make observations from their own cell phones or computers at any time, and they may do so on nights or weekends when there is no facilitator to help them interpret what they are seeing happening in the biological system. These real-time observations are analogous to looking directly through a microscope. Students should capture and log their observations for later use, i.e. keep an elementary lab notebook.

#### 3.1.4 Phase 4: data analysis

**Figure 3:**
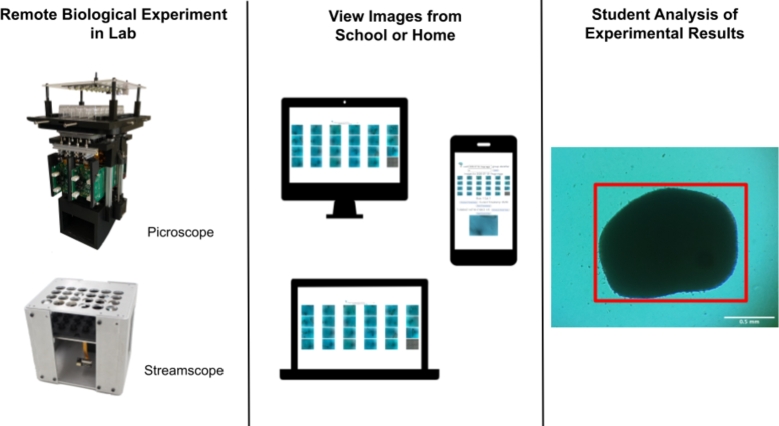
**Remote experiment workflow.** Data is recorded in the remote lab using the Pircoscope or Streamscope, viewed from personal devices, and analyzed by the students.

Finally, the students compile their observations and analyze their data. In addition to live monitoring, time-stamped historical data is also accessible. This allows students to revisit earlier time points to make direct measurements for comparative analysis or make additional observations. Data analysis and the related computer skills required for this are a critical component of modern scientific research that is typically under-emphasized in early college biology coursework [42]. By providing students with simple-to-use analysis tools, students are empowered to look closer at what is occurring and draw more interesting conclusions. For an image-based experiment, this can be accomplished with software that allows students to annotate timestamped images in the data set such that features can be visually tracked over time, and students can note observations on them (Fig. 3).

#### 3.1.5 Phase 5: presentation

When the experiment has concluded and the data has been analyzed, the students present their findings. They should offer a scientific conclusion based on the data and then defend their analysis against questions asked by the instructors and their peers. Presentations can come in many forms, including live slide show presentations, video production, or written reports. An example of a scientific presentation outline is provided in Supplemental Note 5. This mirrors how new science is reported by professionals working in the field and challenges the students to consider the most effective ways to communicate science.

### 3.2 Remote PBL program is adaptable and scalable

We ran four experiments with different user groups to evaluate the flexibility of our framework and possible experiments we can run. These experiments allowed us to iterate on the technology, obtaining user feedback and adapting the technique accordingly. We coordinated with local teachers to ensure that the projects related to real-world problems of interest to the students. These experiments were hosted in San Francisco, CA, while students interfaced with them in near real-time from their homes. Experiment details can be found in Table 1.

**Table 1:** Summary of user studies performed.

Experiment Theme	Biological Sample	Experiment Condition	Number of Users	Geographical Area	Distance from the experiment
Effects of caffeine on development	Zebrafish embryos	Caffeine	12	Salinas, CA, USA	170 Km.
Effects of agriculture byproducts on development and physiology	Zebrafish embryos	Ammonium nitrate, nicotine and caffeine	20	Salinas, CA, USA	170 Km.
Biocompatibility of nanoparticles	Zebrafish embryos	Gold and Graphene Nanoparticles	20	Bolivia, Mexico, Peru, Brazil, and Spain	Up to 9,700 Km.
Toxicity of chlorine dioxide	Zebrafish embryos	Chlorine dioxide	131	Bolivia: Santa Cruz de la Sierra, Cochabamba, La Paz, and El Alto	Up to 8,700 Km.

*Study 1: Alisal High School (proof of concept, single drug)*

As caffeine consumption is higher in student groups compared to the general population [43], we focused our first study on the effects of caffeine exposure on organism development. Students in the AP Biology course at Alisal High School in Salinas, CA observed the effects of three different caffeine concentrations in developing zebrafish embryos. Observations included effects in the whole body, such as movement and twitching, and effects on specific organs, such as the heart. This was our first user study and was important in determining the feasibility of the program with a relatively simple experimental condition

*Study 2: Alisal High School (multiple drugs)*

**Figure 4:**
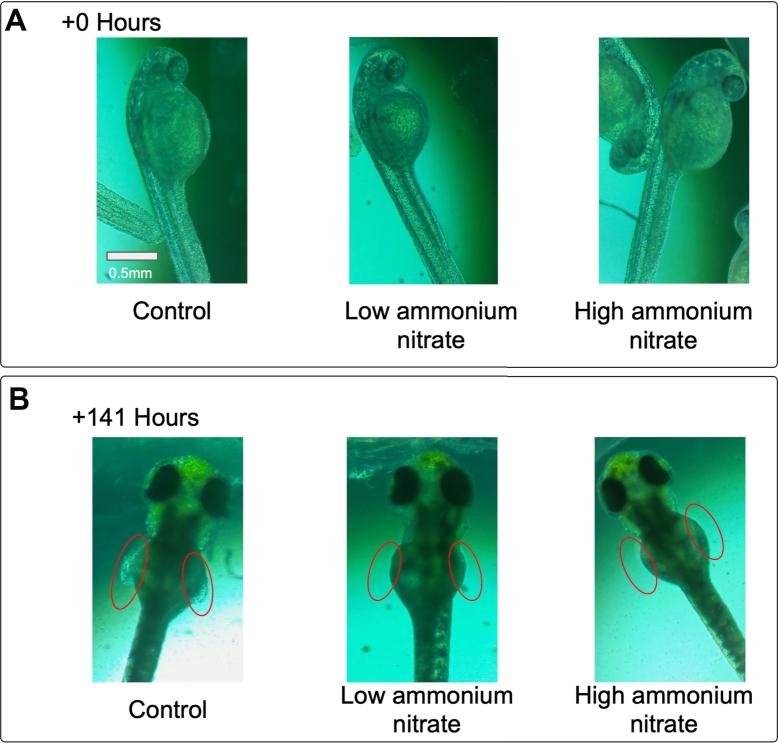
**Context-informed PBL using whole organisms.** Students tracked the effects of chemicals, such as ammonium nitrate, in the development of zebrafish embryos. Representative images over a 141 hours show that ammonium nitrate affects fin development. A) Example of zebrafish embryos at the beginning of the experiment. B) Example of zebrafish embryos after 141 hours shows a delay in fin development in low and high concentrations of ammonium nitrate.

A second user study with a new cohort of AP Biology students also from Alisal High School focused on the effects of common byproducts of agricultural activities. We focused on three chemicals: 1) ammonium nitrate, a chemical commonly used in fertilizers [44], 2) nicotine, which has historically been used as a pesticide and is the chemical basis of several modern neonicotinoid pesticides [45], [46], and 3) caffeine, a contaminant found in cultures of several plants and crops [47]. The students observed the effects of these chemicals in the developing zebrafish embryo. Descriptions of their observations unveiled novel discoveries, such as a delay in fin development in zebrafish exposed to ammonium nitrate (Fig. 4). Measuring the effects of multiple drugs increased the complexity involved and helped to evaluate any pain points that may arise with more complex experiments. We found our framework suitable for facilitating this more complicated experiment.

*Study 3: Five Countries (simultaneous multinational access)*

While there are variable degrees of Internet reliability throughout the United States, availabillity of high speed internet is relatively high compared to the rest of the world [48]. To understand if places with less reliable internet could adopt IoT-enabled PBL, we performed a user study with 20 students in five countries: Brazil, Bolivia, Spain, Mexico, and Peru. The study complemented an online outreach activity by a United States-based non-profit organization Science Clubs International, which targets high school and early college students in the named countries. The students used remote microscopy to perform biocompatibility studies of custom-made gold and graphene nanoparticles and determine the optimal concentration of such particles to be used in the bioengineering context. This study demonstrated the flexibility of our program and showed that it can simultaneously serve students in different places around the world with varying levels of connection reliability.

*Study 4: Bolivia (scalability to large user group with limited internet access)*

In our fourth user study, we focused on scalability. We performed a user study complementing a pharmacology college-level course in Bolivia. This course had over 130 students enrolled across four cities in the country: Santa Cruz de la Sierra, La Paz, El Alto, and Cochabamba. Bolivia has the slowest Internet connection in South America, which makes remote education challenging. Indeed, the government and the education sector have often turned to television and radio as means to conduct large-scale remote education in situations such as the COVID-19 pandemic [49]. In this study, the students tested the toxic effects of chlorine dioxide in animal physiology. Chlorine dioxide is a type of industrial bleach that became highly popular in Latin America during the COVID-19 pandemic, as several politicians promoted its use to prevent SARS-CoV-2 infection [50]. This experiment demonstrated that the system performs well with many more users than were involved in our previous studies. The ability for students to view prior data compensated for any temporary gaps in Internet coverage experienced by the users.

### 3.3 Remote PBL is as effective as in-person PBL at increasing interest in science

**Figure 5:**
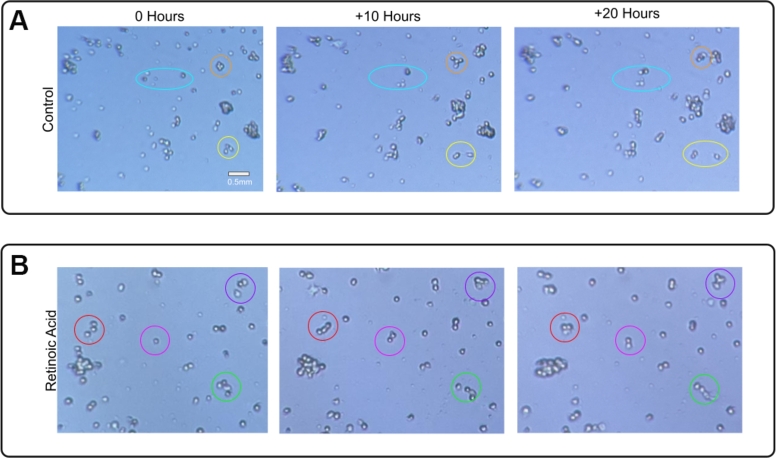
**Studying neuroblastoma cells in the classroom.** The students used IoT-enabled microscopy to understand the effects of drugs, such as retinoic acid, in neuroblastoma cells. Representative images show the tracking of individual cells and cell clusters over 20 hours. A) Example control cells at 0, 10 and 20 hours. B) Cells treated with retinoic acid at 0, 10 and 20 hours.

Our first 4 user studies validated the technology and education approach. To understand the effects of remote PBL education on students' attitudes towards the sciences, we performed another user study. The study participants were first year General Biology students at the Catholic University of Bolivia in Santa Cruz de la Sierra, Bolivia. Santa Cruz de la Sierra is an agricultural hub in Bolivia [51]. Therefore, the project focused on a common issue resulting from fertilizer exposure: the development of childhood cancer [52]. The students studied neuroblastoma, which has been strongly associated with children growing up in agricultural areas [53], and investigated the effects of three drugs on neuroblastoma cell lines (Fig. 5). The three drugs selected were: retinoic acid [54], neurodazine [55], and primocin [56]. The experiment was hosted on a Picroscope in a lab in Santa Cruz, CA. The students compared the effects of each drug with a no-drug control over 5 days. They split into groups and tracked individual cells' migration, division, differentiation, and survival. Groups then discussed their results with their peers and formulated conclusions from their gathered data: the students found a strong effect on cell proliferation and survival after retinoic acid treatment, altered cell morphology following neurodiazine treatment, and no effect from primocin treatment. The students presented their results at their university science fair (Supplemental Figure 1).

At the end of the program, we surveyed the students' attitudes towards STEM and our remote PBL program. The survey questions had all previously been used to assess enthusiasm for STEM after an in-person biology PBL course with another set of Bolivian students [1], allowing us to make comparisons between the remote and in-person PBL courses done with these two cohorts.

We first assessed their attitudes towards science (survey questions included in Supplemental Table 1). On questions relating to overall enthusiasm for science and interest in science careers, the students all responded positively. The one question where the group answers differed was “hard work will help me be successful in science”, where 67% of the remote cohort responded “strongly agree” compared to 90% in the in-person cohort (Table 3 and Supplemental Figure 2A). On questions regarding feelings toward the program they participated in, all participants responded similarly, reporting positive feelings towards these programs (Table 3 and Supplemental Figure 2A).

These results show that among students with similar attitudes towards science, remote PBL is as effective as in-person PBL at increasing interest in science, and that the students enjoyed participating in the remote project.

### 3.4 Remote PBL leads to comparable understanding of the scientific method to in-person instruction

To address whether our remote PBL approach leads to similar learning outcomes as in-person instruction, we tested a group of students in Bolivia with a fully in-person section of the same course. The students were tested on six questions related to the scientific method, including experimental design, data analysis, and outlier identification (Supplemental Note 6). We found that the remote PBL students scored better on the test compared to in-person students (Table 3 and Supplemental Figure 2B, in-person = 76.96 +/- 22.35%, n = 24; remote= 83.17 +/- 19.45%, n = 17; Mann Whitney test. p <0.0001), suggesting that remote experimentation is as effective at teaching the scientific method compared to in-person instruction.

### 3.5 IoT-enabled PBL positively affects different Latinx populations

To investigate how our remote PBL approach impacted different populations of Latinx students, we ran an additional study with a new group of students to compare with our previous data from students in Bolivia. This study was done with AP Biology students from Alisal High School in Salinas, California. Like Santa Cruz de la Sierra, Bolivia; Salinas's primary economy is based in agriculture, and the region has a population that is majority Latinx. [57]. Due to their curricular equivalency, in the United States, AP Biology courses are used to substitute first-year college-level General Biology courses [58]. The Bolivian education system does not have an AP Biology equivalent course in high school, and students are first exposed to complex concepts in cellular and molecular biology in their first year of college. Given the similarities between curricula, we implemented the same neuroblastoma experiment (Fig. 5) with the California group and performed the same supplemental lectures (Table 2)

**Table 2:** Titles and learning outcomes for each lesson taught during the PBL program. Example activities for select lessons can be found in the supplement materials section.

Lesson Title	Learning Outcomes
Scientific Method and Doing Science	1. List and describe the components of the scientific method. 2. Apply the scientific method in an activity.
Central Dogma of Molecular Biology	1. Describe the components of the central dogma of biology. 2. Discuss applications of the central dogma of biology.
Data in Biology	1. Describe the different types of data that can be generated from a biological experiment. 2. Determine how to generate, analyze, and interpret biological data.
Model Organisms	1. Explain what a model organism is and why model organisms are used in biomedical research. 2. Described how scientists can use model organisms to answer specific research questions.
Cell Signaling and Communications Between Cells	1. List the types of cell communication and describe how it occurs via cell surface receptors. 2. Discuss what morphogens are, how they work, and how they affect cell identity.
Microscopy	1. Describe what types of microscopes exist and why scientists use them. 2. Access live data from the Picroscope and explain how you will use the Picroscope for your experiments.
Experimental Design	1. Create a hypothesis you will test during your experiment and explain the rationale behind it. 2. Generate an experimental plan.
Performing Experiments Designed by Students	1. Collect data following experimental plan. 2. Record observations in experimental plan worksheet.
Reflections and Data Analysis	1. Compile and analyze data from the experiment. 2. Describe the outcome of the experiment and the biological meaning of this outcome.
Science Communication and Presenting Your Data	1. Explain why it is important to communicate your findings as a scientist. 2. Determine the most important findings from your experiment.
Student Presentations	1. Communicate your results in a presentation for the class.

**Table 3:** Comparison of students' level of agreement with questions and statements used to assess enthusiasm for STEM after in-person and remote PBL courses. In person PBL data is from Ferreira et al., 2019 [1]. Cohort sizes: In person PBL n = 92, Remote PBL n = 18. Mann Whitney test. Comparison of the mean score received on a test related to the scientific method, administered following the remote or in-person PBL course at the Catholic University of Bolivia. Cohort sizes: In-person PBL n = 24, Remote PBL n = 17. Wilcoxon Signed Rank test. All significance tests were two-tailed.

Statement/Question	Remote Versus In-Person p-value
Science is exciting.	>0.05
I enjoy participating in science projects.	>0.05
I would like to pursue a career in science.	>0.05
I enjoy solving scientific problems.	>0.05
Hard work will help be successful in science.	0.0186
Do you want to become a scientist?	>0.05
Do you want to keep learning more about science?	>0.05
Did our program increase your interst in science?	>0.05
Would you participate in similar programs?	>0.05
*Scientific Method Test*	
Overall score	<0.0001

Like the Bolivian students, the California students tracked the effects of retinoic acid, neurodazine, and primocin on neuroblastoma cells and observed changes compared to the no-drug controls. Similar to the Bolivian case study, the images were taken hourly over a 5-day period and the students analyzed the data in groups. However, unlike the Bolivian user study in which students presented their results at a local science fair, the students in California decided to record videos to disseminate their discoveries over social media (Supplemental Video 2).

After completing their respective courses, we anonymously surveyed the students with two different instruments for quantifying STEM identity, or the level to which STEM interest and ability forms a part of one's personal identity. These instruments are: The STEM Professional Identity Overlap (STEM-PIO-1) and the Role Identity Survey in STEM (RIS-STEM). STEM-PIO-1 is a single-item survey that measures the self-perceived overlap of students with STEM professionals [59]. This instrument has previously been used to differentiate between students in STEM and non-STEM majors and infer desired future STEM identity [59]. RIS-STEM is a 26-question survey designed to measure several aspects of personal STEM identity, including competence in STEM, interest in STEM, self-recognition in STEM, and recognition by others in STEM [60]. Unlike the STEM-PIO-1 instrument, which measures the overall student identity [59], the RIS-STEM instrument disentangles many aspects of STEM identity, such as support by others [60], which can shed light onto various identity differences between groups of students. We then asked the students how participating in our remote PBL program affected their answers to the these questions.

**Figure 6:**
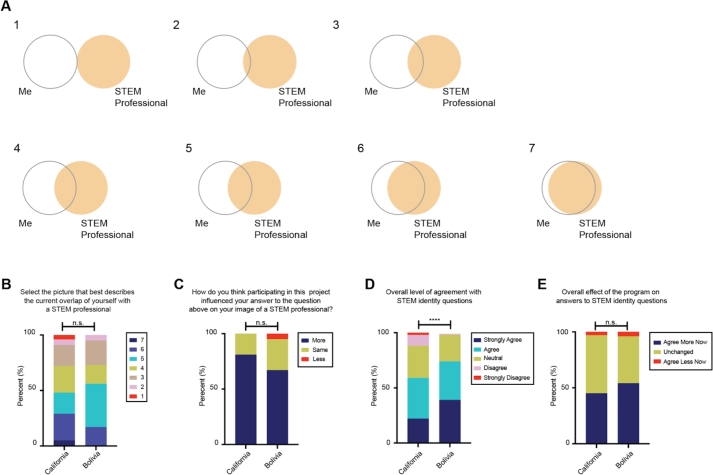
**IoT-enabled PBL positively affects STEM identity in different Latinx cohorts.** (A) Diagrams used in the STEM-PIO-1 instrument to assess STEM identity. Students are asked to select the picture that best describes their overlap with a STEM professional. (B) Level of agreement with the question “Select the picture that best describes the current overlap of yourself with a STEM professional”, referring to the image in A. (C) Impact of the remote instruction project on the answer to the question in B. (D) Overall level of agreement with 26 questions to assess STEM identity in the RIS-STEM instrument. (E) Overall impact of the remote instruction project on the level of agreement with statements in D. Cohort sizes: California n = 21, Bolivia n = 18. Mann Whitney test. **** = p <0.0001; n.s. = not significant.

Responses to the STEM-PIO-1 indicate similar self-perceived overlap with a STEM professional between the two groups (p >0.05) (Fig. 6A, B). These results are comparable to those of students in STEM majors, as reported previously [59]. When surveyed, the majority of students in both groups reported that participating in this program had a positive effect on their STEM-PIO-1 responses (Fig. 6C).

**Figure 7:**
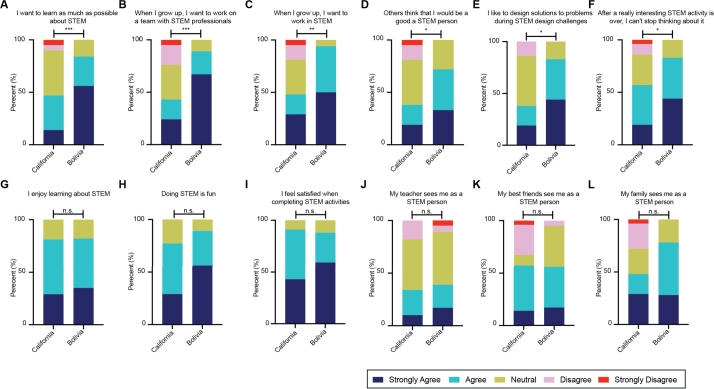
**Different Latinx student groups have distinct STEM identities.** (A-L) Students' answers to the questions in the RIS-STEM instrument. (A-F): Statistically significant answers between student cohorts in California and Bolivia: A) I want to learn as much as possible about STEM, B) When I grow up, I want to work on a team with STEM professionals, C) When I grow up, I want to work in STEM, D) Others think that I would be a good a STEM person, E) I like to design solutions to problems during STEM design challenges, F) After a really interesting STEM activity is over, I can't stop thinking about it. (G-L): Sample statistically not significant answers between Californian and Bolivian students: G) I enjoy learning about STEM, H) Doing STEM is fun, I) I feel satisfied when completing STEM activities, J) My teacher sees me as a STEM person, K) My best friends see me as a STEM person, L) My family sees me as a STEM person. Additional answers in Supplemental Figure 4. Cohort sizes: California n = 21, Bolivia n = 18. Mann Whitney test. *** = p <0.001; ** = p <0.01; * = p <0.05; n.s. = not significant.

Unlike the STEM-PIO-1 results, we observed that the Bolivian students agreed significantly more overall with the RIS-STEM survey questions compared to the students in California (Fig. 6D). This difference can be attributed to the Bolivian students' increased agreement with statements that involved doing and learning more about STEM in the future compared to the California students (Fig. 7, top). For instance, 56% of Bolivia students selected “strongly agree” to “I want to learn as much as possible about STEM”, compared to only 14% of California students (Mann Whitney test, p = 0.0038) (Fig. 7, top). The remaining RIS-STEM questions received similar answers from both cohorts (Fig. 7, bottom and Supplemental Figure 3).

Despite their differences, the Bolivia and California cohorts both positively evaluated their participation and the impact of our program on their STEM identity (Fig. 6E and Supplemental Figure 4). Together, these results indicate that both groups feel similar overlap with STEM professionals, but exhibit differences in their desire to continue studying STEM. Still, our remote PBL program positively impacted STEM identity within both cohorts, regardless of differences in their attitudes towards science.

### 3.6 Student comments indicate positive experiences with our remote PBL program

Beyond the multiple-choice sections of the survey, students were also given questions with free response answers. The ability for the students to easily use the imaging viewer is crucial for them to observe and analyze the results of their experiment. In the survey, we asked the students for feedback on the imaging viewer, and received overwhelmingly positive results:

*“The imaging viewer was very easy to access and after being taught on how to understand it, it was very easy to look for the data we wanted and find clear pictures to present.”*

*[California Student]*

*“The imaging viewer was very user-friendly. I had no trouble clicking back and forth through pictures.”*

*[California Student]*

*“It was very easy to use the website to access the images.”*

*[Bolivia student, translated from Spanish]*

We also asked the students whether they enjoyed being able to watch a live experiment in near real time, and if so, what they liked about it. The students largely appreciated the live aspect of the experiment:

*“Yes, I did enjoy watching a live experiment in almost real time because it felt like it was in a real lab and I was able to see how it was that an experiment is set up.”*

*[California Student]*

*“It was really interesting being able to come back a day later to see how much the cells had changed and making discoveries in real time rather than looking at something that's already been observed in the past.”*

*[California Student]*

*“I loved it! It was very interesting to run a long-term project, as we had the opportunity to constantly collect data and progressively get new results that we could see in real time.”*

*[Bolivia student, translated from Spanish]*

*“I liked being able to work with samples that are thousands of kilometers away, I feel that it is something that opens the doors for us to have a more entertaining and broader education.”*

*[Bolivia student, translated from Spanish]*

One of the goals of the project is to increase students' knowledge and skills in scientific research. To achieve this goal, students were exposed to techniques in experimental design, data collection, and data analysis throughout the course and in their final project. When asked to report “the most interesting or useful thing you feel you learned in a post-course evaluation”, several scientific skills were mentioned:

*“The most useful thing I feel I learned was how to use a microscope and collect data properly.”*

*[California Student]*

*“The most useful thing I learned was how to analyze the data and how to practice coding.”*

*[California Student]*

*“I learned how to collect data and apply it to a real situation.”*

*[California Student]*

The students also enjoyed the visual nature of the project, with one student writing:

*“The most interesting thing I learned was how to visually see and recognize normal cells and cancer cells. Most useful thing I learned was how to properly analyze an image.”*

*[California Student]*

Their interest in the project topic, cancer cell growth, was also commonly mentioned throughout the students' evaluation responses. Some more examples of their responses to the same question as above, “What was the most interesting or useful thing you feel you learned?” included:

*“The most interesting thing I learned was how cancer cells look over time because I didn't know how they looked before.”*

*[California Student]*

*“The most interesting thing I learned was about the three different types of medicines that can be used against cancer cells and how those affected the growth of cancer cells, it was also interesting to look over the images of the cancer cells and see them close up to analyze them.”*

*[California Student]*

*“I feel like I have better knowledge about cell functions and how different factors can affect the body. Going into depth about cell death and growth was mind-blowing and made me much more aware of what happens in our body.”*

*[California Student]*

*“How neuroblastoma cells behave with certain drugs and how that can be used to improve people's lives in the future.”*

*[Bolivia student, translated from Spanish]*

*“Seeing how drugs act at a microscopic level in cells.”*

*[Bolivia student, translated from Spanish]*

These comments suggest that the IoT-enabled imaging technology is sufficient to expose students to complex experiments while fostering an enjoyable and interesting experience and that the Picroscope imaging viewer is easy to use for high school students. These responses also reveal that the relevance of the scientific topic is an important factor for generating interest in the experiment and should be considered for future projects.

## 4 Discussion

Despite significant investments in education, Latinx people continue to be underrepresented in the sciences. Education technology ‘megaprojects’ such as the One Laptop per Child project and EdX have shown that access to technology alone is ineffective at motivating underrepresented students [61], [62]. Therefore, novel approaches that can integrate scalable technologies with proven successful teaching methodologies are needed to target students effectively [63].

We used IoT-enabled microscopy to complement high school and college biology courses for student populations in the United States, Bolivia, Brazil, Spain, Colombia, and Mexico. Our framework allows for the instruction of the students in their native language using PBL and was adapted to a variety of real-world projects, such as caffeine consumption in students, agriculture chemical exposure, and pharmacology.

A fundamental issue in social sciences studies is the assumption that a group studied is representative of the entirety of the population [64]. Given the direct applicability of education studies toward the development of interventions and policies, it is imperative to understand the diversity of the group to be analyzed. Latinxs are not a homogeneous group, and different cultural characteristics can play an important role in learning [65].

Historically, most education studies in Latinxs have taken place in the United States and Puerto Rico, and the results have been extrapolated to other Latinx groups [64], [65]. Performing multinational PBL education studies is not trivial, as countries have different regulations, academic priorities and calendars that can interfere with the execution of the studies [66]. As a consequence, few studies have assessed the outcome of given interventions across borders and into continental Latin America [67], [68], [69], [70]. Moreover, the majority of these studies have focused on neighboring countries, particularly in STEM courses that required an experimental component [67], [68], [70]. IoT-enabled technologies can provide a unique opportunity to overcome these limitations and reach students throughout the world.

Currently, most studies on education in underrepresented groups are based on relatively small geographical locations. For example, studies in Latinx populations tend to be based on immigrants or immigrant-descent students living in the United States [71], [72]. Yet, previous work has shown that, at least for biology education, results of studies in Latin America differed significantly from expected [1]. However, direct comparative studies between groups are difficult due to logistical and systematic issues, such as language barriers, differences in teacher training, and variations in academic calendars. Here, we took advantage of IoT-enabled microscopy to compare the effects of remote PBL between groups. For example, we found that Bolivia students had stronger STEM identities than their California counterparts (Fig. 6D, Fig. 7). As strong STEM identity is a predictor of future STEM career choice [73], understanding the roots of these differences is important to better target education approaches that can increase diversity in STEM.

A unique aspect of this remote PBL paradigm is that it allows students to run experiments using materials that would otherwise be inaccessible due to their hazardous potential or the difficulty of transporting them to remote locations. For example, the AP Biology and college general biology curricula include content on mammalian cellular biology [74]. Yet, experiments in those courses have been limited in scope, focusing on microbiology or plant biology [75]. Students are usually not exposed to experimental mammalian cell and tissue culture until upper division college courses [76]. Given the differences in cellular biology between life kingdoms, there is a disconnect between theory and practice in high school and lower-level college biology courses. We addressed this disconnect by creating a context-informed project centered on the culture of neuroblastoma cells and the effects of various drugs on those cells. These “clinical trials in a dish” expose students to many facets of scientific work and introduce them to career paths in STEM that may have been unknown to them, including biochemistry, analytical chemistry, and bioinformatics. Most surveyed students self-reported that this program positively impacted their interest in science and pursuing scientific careers, and we expect that this will be important for increasing diversity in STEM.

While our program appears effective and engaging for our student participants, we acknowledge certain limitations within the data we are presenting. These limitations motivate the need for more research to better understand and evaluate the effects of programs like ours. The relatively small sample size in our user studies means that our cohorts may not be representative of the general population within the communities we ran programs in. Additional cohort differences in factors like age and economic status may confound comparisons made between groups in different countries. A closer look at the academic impact of our program would give a more clear measure of its benefits. While a subject test administered with a control group was done with the Bolivian cohort to evaluate learning outcomes, the bulk of our gathered data concerns the effects of the program on social metrics like engagement, satisfaction, and identity.

Future research would do well to address these limitations by running studies with many groups within a particular country at different institutions with a diverse set of students spanning various age groups and stages in their education. Furthermore, greater evaluation of learned content could be done by looking at resulting class grades, as well as having more comprehensive exams done before and after the program for both participants and a control group.

Our future vision for the PBL remote experimentation framework is to create a community environment with massive open online experimentation. Many science labs and classrooms can collectively enroll in a session to connect students worldwide while still having them benefit from learning in a small classroom. Each session would focus on a specific experimental question. All students would crowd-source ideas about the procedure, launch experimental conditions, share and analyze data, and present their findings in a culminating event. Stay informed on future updates at https://braingeneers.gi.ucsc.edu/.

On the technical side, a future direction would be to connect more sensors and controllers to each experiment, such as devices that measure electrical activity, automated drug delivery, and light stimulation. Another exciting future direction would be to improve the software visualizations. An immersive online environment could be created in 3D through AR/VR (Augmented Reality/ Virtual Reality), where students use their phones to manipulate the experimental equipment and models. The manipulations they perform in a VR environment could change experimental devices in the lab connected through IoT. We are eager to continue exploring the potential of this technology to facilitate ever-improving educational experiences for everyone.

## Declarations

### Author contribution statement

Pierre V. Baudin; Raina E. Sacksteder: Performed the experiments; Analyzed and interpreted the data; Contributed reagents, materials, analysis tools or data; Wrote the paper.

Atesh K. Worthington: Performed the experiments; Analyzed and interpreted the data; Contributed reagents, materials, analysis tools or data.

Kateryna Voitiuk; Ryan N. Hoffman; Matthew A.T. Elliott; David F. Parks; Rebecca Ward; Sebastian Torres-Montoya; Finn Amend; Natalia Montellano Duran; Paola A. Vargas; Guadalupe Martinez; Sandra M. Ramirez; Lucia Elena Alvarado-Arnez; Arnar Breevoort; Tallulah Schouten: Performed the experiments.

Victoria T. Ly; Drew Ehrlich; Yohei M. Rosen: Performed the experiments; Contributed reagents, materials, analysis tools or data.

Sri Kurniawan; David Haussler; Mircea Teodorescu: Conceived and designed the experiments.

Mohammed A. Mostajo-Radji: Conceived and designed the experiments; Wrote the paper.

### Funding statement

This work was supported by Schmidt Futures (SF857) to D.H. and M.T.; 10.13039/100000051National Human Genome Research Institute (1RM1HG011543) to D.H. and M.T.; 10.13039/100000001National Science Foundation (NSF2134955) to D.H. and M.T.; D.H. is a Howard Hughes Medical Institute Investigator.

### Data availability statement

Data included in article/supp. material/referenced in article.

### Declaration of interests statement

The authors declare the following conflict of interests: P.V.B., K.V., V.T.L., Y.M.R., D.H. and M.T. have submitted patent applications related to Internet-of-Things-enabled microscopy. M.A.M.-R. is a cofounder of Paika, a company for remote people-to-people interactions. The authors declare no other conflicts of interests.

### Additional information

Supplementary content related to this article has been published online at https://doi.org/10.1016/j.heliyon.2022.e11596.

No additional information is available for this paper.

## Supplementary material

The following Supplementary material is associated with this article:

IoT-enabled education paper supplemental materials Supplemental Figure 1: Bolivian students' presentations. Supplemental Figure 2: Comparison of students' level of agreement with questions and statements used to assess enthusiasm for STEM after in-person and remote PBL courses. Supplemental Figure 3: Answers of student cohorts to the RIS-STEM instrument. Supplemental Figure 4: Different student cohorts report similar effects to IoT-enabled PBL. Supplemental Table 1: Student Survey Questions. Supplemental Note 1: Data in Biology Activity. Supplemental Note 2: Model Organisms Activity. Supplemental Note 3: Experimental Design Activity. Supplemental Note 4: Performing Experiments Activity. Supplemental Note 5: Science Communication Activity. Supplemental Note 6: Scientific Method Test. IoT-enabled education paper supplemental materials

Baudin et al Supplemental Video 1 Remote Image Viewer Tutorial. Baudin et al Supplemental Video 1

Baudin et al Supplemental Video 2 Alisal High School students' presentations reporting their results. Baudin et al Supplemental Video 2
